# Supplementary material for: Clarifying the meaning of a positive living environment in nursing homes: A scoping review protocol
Source: PLoS One. 2025 May 29;20(5):e0324336. doi: 10.1371/journal.pone.0324336 (PMC12121755; doi:10.1371/journal.pone.0324336)
Supplement: Attachment — Submitted filename: Response to Reviewers.docx [file pone.0324336.s003.docx]

PLOS ONE

Editorial office

***27 February 2025***

**R****ebuttal letter manuscript – “Clarifying the meaning of a positive living environment in nursing homes: a scoping review protocol”**

Dear Editor-in-Chief, dear Prof. Dr. Chenette,

Dear members of the Editorial Board,

Thank you for the opportunity to revise and resubmit our manuscript entitled *Clarifying the meaning of a positive living environment in nursing homes: a scoping review protocol* for publication in the Journal of PLOS ONE.

We have carefully considered each point raised by the academic editor and reviewers. On the following pages, we provide a detailed response to each comment and outline the changes we have made to improve our manuscript.

We believe that the revisions and clarifications provided in response to the reviewers' comments have strengthened our manuscript. We hope that it now meets the standards for publication in the Journal of PLOS ONE.

We hope you find our revised manuscript suitable for publication and look forward to hearing from you at your earliest convenience.

Yours sincerely,

On behalf of all authors,

Mieke Deschodt

Journal requirements

1. When submitting your revision, we need you to address these additional requirements. Please ensure that your manuscript meets PLOS ONE's style requirements, including those for file naming**.** The PLOS ONE style templates can be found at:

<https://journals.plos.org/plosone/s/file?id=wjVg/PLOSOne_formatting_sample_main_body.pdf> 
<https://journals.plos.org/plosone/s/file?id=ba62/PLOSOne_formatting_sample_title_authors_affiliations.pdf>

*Thank you for bringing this to our attention. We have adjusted the manuscript, including file naming, to necessary style requirements.*

2. We note that you have indicated that there are restrictions to data sharing for this study. PLOS only allows data to be available upon request if there are legal or ethical restrictions on sharing data publicly. For more information on unacceptable data access restrictions, please see <http://journals.plos.org/plosone/s/data-availability#loc-unacceptable-data-access-restrictions>.

*Since this manuscript is a protocol paper, no datasets were generated or analyzed. For the scoping review, the data extraction forms will be provided as supplementary material in the forthcoming publication. To clarify this, we have updated our data availability statement in the submission form.*

Before we proceed with your manuscript, please address the following prompts:

a) If there are ethical or legal restrictions on sharing a de-identified data set, please explain them in detail (e.g., data contain potentially identifying or sensitive patient information, data are owned by a third-party organization, etc.) and who has imposed them (e.g., a Research Ethics Committee or Institutional Review Board, etc.). Please also provide contact information for a data access committee, ethics committee, or other institutional body to which data requests may be sent.

b) If there are no restrictions, please upload the minimal anonymized data set necessary to replicate your study findings to a stable, public repository and provide us with the relevant URLs, DOIs, or accession numbers. For a list of recommended repositories, please see

<https://journals.plos.org/plosone/s/recommended-repositories>. You also have the option of uploading the data as Supporting Information files, but we would recommend depositing data directly to a data repository if possible.

Review Comments to the Author

Reviewer #1:

The protocol demonstrates several strengths that enhance its suitability for publication. Notably, it effectively integrates scoping review and concept analysis methodologies, ensuring a comprehensive and nuanced exploration of the concept of a positive living environment in nursing homes. The inclusion of perspectives from multiple stakeholders, such as residents, staff, and informal caregivers, strengthens the depth and breadth of the analysis. The use of well-established frameworks, such as Levac et al.’s scoping review methodology and Walker and Avant’s concept analysis approach, provides a robust methodological foundation. Additionally, the registration of the study on the OSF and the inclusion of the PRISMA-ScR checklist reflect adherence to transparency and reporting standards.

However, there are a few areas where the protocol could benefit from further refinement. While the decision to include only studies published in English and Dutch is acknowledged, a more explicit justification for this limitation would help address potential selection bias. The authors should also elaborate on strategies to mitigate interpretation bias, such as triangulation or external validation methods. Given the cultural variability in perceptions of a positive living environment, a discussion of how the findings might generalize to diverse cultural contexts would further enhance the protocol’s applicability. Lastly, while dissemination plans are mentioned, providing more detail on how the findings will influence policy or practice in nursing home settings would strengthen the discussion and highlight the study’s potential impact.

These adjustments will help refine the protocol and ensure its relevance and rigor in contributing to the field of nursing home care

*Dear reviewer, thank you for your valuable suggestions.*

*Regarding the suggestion about the language restrictions, this decision was primarily based on the language proficiencies of the research team. We acknowledge that the focus on English and Dutch literature can introduce a selection/cultural bias. We will include an overview of the countries where the primary research results stem from in the data analysis of the scoping review, meaning we will be transparent about the cultural contexts of the included studies. Further, our findings will account for the cultural variability in perceptions of a positive living environment, by using an iterative approach during data extraction and analysis (see additional section in manuscript, included below). This allows us to distinguish between core elements and context-specific elements, ensuring that cultural influences are embedded in the analysis rather than treated as separate factors.*

*To mitigate interpretation bias, we will hold multiple group discussions within the research team (peer review). The research team consists of members with diverse backgrounds including nursing, psychology, and expertise in qualitative research. This in combination with the iterative process of data extraction and analysis, ensures that no relevant information is overlooked. By integrating multiple perspectives and staying close to the original data — which itself reflects the views of staff, residents, and informal caregivers—, we aim to minimize interpretation bias while maintaining flexible and open to emerging insights.*

*We clarified these elements in the methodology and added justification for these limitations in the following sections:*

*Data extraction section (page 7, lines 162-170)*

*‘A customized data extraction form will be developed to extract all relevant data from each included study. …*

*To facilitate this process, we will first create one-page conceptual sheets for each individual study.* *In these sheets we will extract the most relevant information while considering the specific context of each study. Through this analysis, we will identify relevant elements and classify them as defining attributes, antecedents, consequences or empirical referents. Consequently, the data extraction and synthesis phases will be iterative.*

*The data extraction form will be tested using five included full-text papers.* *Given the iterative nature of data extraction and synthesis, the form will be refined, updated, or clarified as needed until all authors reach a consensus on the final version.’*

*Synthesis and presentation of results section (page 9, lines 192-200):*

*‘The foundation for this analysis will be the one-page conceptual sheets developed for each included study (see ‘Data extraction’ section). These conceptual sheets will be used to conduct a cross-case analysis guided by the Qualitative Analysis Guide of Leuven (QUAGOL) [25]. Through groups discussions with the research team, we will refine these conceptual sheets based on emerging insights, to ensure that all relevant information is captured. This approach will help us systematically differentiate between the various elements of the concept analysis while also distinguishing between core aspects and context-specific elements, ensuring that cultural influences are embedded in the analysis rather than treated as separate factors. This approach will help to refine, evaluate and achieve consensus regarding the findings.’*

*Discussion section (page 11, lines 247-258):*

*‘Further, based on the language proficiencies of the research team members, we will only include Dutch and English papers. This might introduce cultural bias, meaning that our findings may not fully represent perspectives from all cultural contexts. To address this, we will report the country of each included study, providing insights and transparency regarding the cultural scope of our findings. Additionally, the iterative process of data extraction and analysis, using the conceptual sheets and conducting cross-case analysis, ensures that our findings will reflect both generalizable elements and context-specific nuances. This allows us to identify the shared core themes while acknowledging cultural differences. By maintaining flexible and open to emerging insights throughout the analysis, we aim to enhance the rigor and comprehensiveness of our findings. Furthermore, the involvement of multiple reviewers with diverse backgrounds, including nursing, geriatrics, psychology and qualitative research expertise, ensures a broad range of perspectives and minimizes individual bias and interpretation bias as much as possible.’*

*Regarding the impact of our findings on policy and practice, we outlined in the discussion section how each component of the concept analysis can inform policy development and practical implementation. We have expanded this section to further emphasize the connection between our findings and their applicability to nursing home policies and care strategies.*

*We adapted the discussion section accordingly (page 10, lines 217-232) :*

*‘We will integrate Walker and Avant’s concept analysis methodology with Levac and colleague’s scoping review methodology [14, 15], as the latter lacks clear guidelines for data extraction and qualitative analysis. This integration allows us to thoroughly explore and clarify our concept of a positive living environment, which in turn will inform policy, researchers and healthcare workers to develop targeted interventions and quality improvement projects in nursing homes. More specifically, the defining attributes will indicate which organizational, team or process related components can be improved to create or maintain a positive living environment, whereas the antecedents will provide guidance on context-specific implementation strategies tailored to the needs of staff and residents, such as the necessary training programs for these attributes to manifest. Furthermore, the identification of the consequences will help set measurable goals and objectives for future improvements, while the empirical referents can enable the development of tools and methods to evaluate and monitor the quality of the living environment in nursing homes, enabling continuous progress tracking and necessary adjustments. So by using the combined methodology of scoping review and concept analysis we can establishes a clear framework for future studies as well as for policy decisions. This ensures that efforts to foster positive living environments are based on well-defined, evidence-based and measurable criteria.’*

*We hope these adjustments strengthen the manuscript and address your concerns.*

Reviewer #2:

The manuscript is well-prepared and establishes a strong foundation for the scoping review. However, minor revisions are needed to justify its methodological choices and address potential biases. Here's my suggestion:

*Dear reviewer, thank you for your valuable suggestions. Please find below our answers and adjustments we have carried out based on your feedback.*

1. The exclusion of studies older than 20 years and non-English/Dutch papers may limit the comprehensiveness. Justify these exclusions more robustly.

*We elaborated on the exclusion criteria of studies older than 20 years in the discussion section (page 10, lines 233-242):*

*‘Nonetheless, this scoping review will have some limitations. The scoping review will solely focus on literature published within the last 20 years. While this excludes historical perspectives, the review aims to provide insights into the most relevant and current practices and policies in nursing home settings. Significant demographic shifts such as rapid population aging, the rising prevalence of chronic diseases, and the decreasing availability of family caregivers due to higher workforce participation among women and declining birth rates have reshaped the landscape of the nursing home [1]. Recent literature more accurately reflects these evolving challenges and needs. By focusing on the past two decades, the review ensures that the findings are aligned with current trends and offer insights into the development of effective interventions and tools that address both present and emerging issues within the nursing home.’*

*The rationale for including only English and Dutch studies is addressed above, in the first paragraph of our response to reviewer 1 (see also page 11, lines 247-258 in the manuscript).*

2. Clarify the rationale for excluding pandemic-specific studies, as they provide relevant insights into positive living environments under stress.

*The decision to exclude studies specifically focusing on the impact of the COVID-19 pandemic on nursing homes was made to maintain a focus on the broader, non-pandemic-specific factors that contribute to a positive living environment. While the pandemic has undoubtedly introduced unique challenges, our aim is to identify the core elements that define a positive living environment under typical conditions. Studies focusing on pandemic-specific issues such as lockdowns, visitor restrictions, and infection control measures may highlight temporary or atypical aspects of nursing home life that are not representative of typical circumstances. By excluding these studies, we seek to gain a clearer and more robust understanding of the fundamental attributes, antecedents, consequences, and empirical referents of a positive living environment. This approach allows us to provide insights that are generally applicable, rather than specific to crisis situations. We acknowledge that this exclusion is a limitation and that future research could explore the impact of pandemics and similar crises on the concept of a positive living environment, building on the findings of this review. However, within the scope of this study, we focus on defining what constitutes a positive living environment in a general sense. During data analysis, we will consider the pandemic's impact by examining potential shifts in the literature published before and after COVID-19.*

*We added this justification in the discussion section (page 11, lines 242-246):*

*‘Related to this, we will exclude studies specifically focusing on the COVID-19 pandemic. While the pandemic introduced unique challenges, our goal is to identify core elements of a positive living environment under typical conditions. Pandemic-related issues may highlight temporary or atypical aspects of nursing home life, limiting generalizability. Yet future research could explore the impact of pandemics and similar crises on the nursing home living environment.’*

3. Address interpretation and selection biases more thoroughly in the discussion.

*For interpretation and selection biases, we have outlined our strategies in the response above, in the first and second paragraph of our response to reviewer 1. Further details are provided in the manuscript’s discussion section (page 11, lines 247-258)*

4. To ensure practical relevance, include a plan for validating findings with nursing home residents, staff, or informal caregivers.

*The findings of the scoping review will mainly be validated with a research team, consisting of members with backgrounds in nursing, relevant expertise in research, and hands-on experience in the nursing home context. Importantly, the primary research included in this review is based on qualitative data collected directly from these stakeholder groups (residents, staff and informal caregivers), meaning that our findings are already grounded in their perspectives. For this specific study, we do not plan to formally validate the results with additional residents, staff, or informal caregivers. However, the scoping review is part of a broader research project in Flanders, were we will conduct qualitative studies, including interviews, focus groups and workshops with various stakeholders, to explore what factors contribute to a positive living environment in Flemish nursing homes for residents.*

*We now mention in the protocol that the scoping review is part of a broader research project (manuscript page 11, lines 260-266)*

*‘Moving forward, the findings of the scoping review will be disseminated through a peer-reviewed publication, as well as presentations at scientific conferences and network events. Furthermore, the scoping review is part of a broader research project in Flanders, where additional qualitative studies, including interviews, focus groups, and workshops with various stakeholders, will explore the factors that contribute to a positive living environment in Flemish nursing homes. These future studies will help contextualize the findings within the Flemish setting, while validating and expanding upon the insights gained from this scoping review. By broadly sharing our results, we aim to contribute to ongoing efforts aimed at improving nursing home residents’ well-being.’*

5. Explicitly outline how findings will inform the development of practical interventions or frameworks.

*Regarding the impact of our findings on policy and practice we refer, to the final paragraph of our response to reviewer 1. We added additional justification based on this suggestion within the discussion section (page 10, lines 217-232)*

*We hope these adjustments strengthen the manuscript and address your concerns.*

Reviewer #3:

Abstract:
Please make the background concise, and present the gap in the literature by presenting other relevant concepts like safe environment, safe space.

*We added relevant concepts in relation to a positive living environment in background part of the abstract:*

*‘Background: Nursing homes provide permanent residence and care for older individuals who can no longer live independently. Despite efforts to create a safe and homelike environment, concerns persist regarding residents’ well-being. Current research often focuses on different aspects, such as quality of life, sense of home or thriving in the nursing home, each only providing a partial understanding of what constitutes a positive living environment. To address this, we will explore what entails the concept of ‘a positive living environment' in nursing homes from the perspectives of residents, staff, and informal caregivers.’*

Introduction:

Present the importance of nursing home. Present relevant epidemiologic/statistical data with regards to aging population, patients of nursing homes, and nursing homes.

*We have incorporated relevant epidemiologic data on the aging population and nursing home residents, including the increasing prevalence of chronic diseases and cognitive impairments, which contribute to a higher care burden.*

*(page 3, lines 45-51):*

*‘The global population is aging rapidly, with the proportion of individuals aged 80 and older expected to double from 4.6% in 2017 to 10.1% by 2050 [1]. As both the total number and proportion of older adults increase, the prevalence of chronic diseases and cognitive impairments is also rising [1]. Consequently, nursing home residents have increasingly complex and demanding care needs. This, in combination with a declining availability of informal caregivers, further intensifies the demand for long-term care facilities, such as nursing homes, to provide permanent residences for older individuals who can no longer live at home [1, 2]’*

Present existing relevant concepts of nursing homes.

*We address the shift in nursing homes types toward a more personalized approach, moving away from a purely clinical care model, to better support residents' well-being, such as nursing homes with small-scale living environments as well as the Green House homes.*

*(page 3, lines 53-57):*

*‘In line with this, healthcare organizations are striving to make nursing homes feel more like a home rather than a clinical facility, as seen in the growing emphasis on providing care in small-scale living environments or Green House Homes [5]. These types of facilities move from a strictly clinical care model to a more person-centered care model meaning they are designed to create a homelike feeling while also offering high-quality care [5].’*

Methodology:

Looking into the purpose and overall aim of the study, I believe that a scoping review is the least appropriate method to use compared to methods like concept analysis of Walker and Avant and integrative review by Whittemore and Knafl or evolutionary methods.

Please review the different types of review using these suggested links:

Systematic review and scoping review:

<https://link.springer.com/content/pdf/10.1186/s12874-018-0611-x.pdf>

Concept analysis:

<https://www.researchgate.net/profile/Judith-Hupcey/publication/14198489_Concept_analysis_in_nursing_research_A_critical_appraisal/links/5654b6c808aeafc2aabc0093/Concept-Analysis-in-Nursing-Research-A-Critical-Appraisal.pdf>

<https://www.researchgate.net/profile/Lisbeth-Fagerstroem/publication/49620753_Rodgers%27_evolutionary_concept_analysis-A_valid_method_for_developing_knowledge_in_nursing_science/links/5a8e79d0a6fdcc808c0f8d22/Rodgers-evolutionary-concept-analysis-A-valid-method-for-developing-knowledge-in-nursing-science.pdf>

Integrative review:

<https://www.researchgate.net/profile/Lisbeth-Fagerstroem/publication/49620753_Rodgers%27_evolutionary_concept_analysis-A_valid_method_for_developing_knowledge_in_nursing_science/links/5a8e79d0a6fdcc808c0f8d22/Rodgers-evolutionary-concept-analysis-A-valid-method-for-developing-knowledge-in-nursing-science.pdf>

*We agree that concept analysis, specifically the Walker and Avant methodology, is the most appropriate approach for analyzing the data in this study. We have chosen to integrate this methodology with a scoping review methodology given that concept analysis, as traditionally applied, does not include a structured, systematic search strategy to identify relevant studies. To address this, we incorporate a scoping review approach, which allows us to conduct a comprehensive literature search using predefined databases and a structured search string. This ensures that we capture relevant studies systematically.*

*Once the literature is gathered, we apply the Walker and Avant concept analysis framework to examine the uses of the concept, the defining attributes, antecedents, consequences, and empirical referents. By combining these two methods, we ensure a rigorous and systematic approach while maintaining the depth of analysis provided by concept analysis. Further details on this approach are outlined throughout the whole manuscript, including in the design section (page 5, lines 91-99).*

*‘Design*

*This review will use the methodological framework described by Lam Wai Shun and colleagues, which combines elements from both the scoping review and the concept analysis methodology [15]. We will use scoping review methodology of Levac and colleagues [18] building upon the framework proposed by Arksey and O’Malley [19] to systematically identify and select relevant publications as well as for numerical analysis. To extract and analyze data for thematic analysis we will use the Walker and Avant concept analysis methodology, which offers structured guidelines for concept clarification by using multiple steps: defining uses of the concept, defining attributes, antecedents, consequences, and empirical referents [14].’*
