# Supplementary material for: Clarifying the meaning of a positive living environment in nursing homes: A scoping review protocol
Source: PLoS One. 2025 May 29;20(5):e0324336. doi: 10.1371/journal.pone.0324336 (PMC12121755; doi:10.1371/journal.pone.0324336)
Supplement: Attachment — Submitted filename: Response_to_Reviewers_auresp_2.docx [file pone.0324336.s004.docx]

PLOS ONE

Editorial office

***14 April 2025***

**R****ebuttal letter manuscript – “Clarifying the meaning of a positive living environment in nursing homes: a scoping review protocol”**

Dear Editor-in-Chief, dear Prof. Dr. Chenette,

Dear members of the Editorial Board,

Thank you for the opportunity to revise and resubmit our manuscript entitled *Clarifying the meaning of a positive living environment in nursing homes: a scoping review protocol* for publication in the Journal of PLOS ONE.

We have carefully considered each point raised by the academic editor and reviewers. On the following pages, we provide a detailed response to each comment and outline the changes we have made to improve our manuscript.

We believe that the revisions and clarifications provided in response to the reviewers' comments have strengthened our manuscript. We hope that it now meets the standards for publication in the Journal of PLOS ONE.

We hope you find our revised manuscript suitable for publication and look forward to hearing from you at your earliest convenience.

Yours sincerely,

On behalf of all authors,

Mieke Deschodt

Review Comments to the Author

Reviewer #2:

The justification for the exclusion criteria is clearly outlined, specifying the focus on English and Dutch studies and the rationale for excluding pandemic-specific research. The methodological rigor is strengthened by integrating the scoping review methodology with Walker and Avant’s concept analysis, ensuring a systematic and in-depth examination of the topic. To mitigate bias, the authors have included steps to address both interpretation and selection biases, enhancing the reliability of the findings. Additionally, the discussion highlights the practical relevance of the research, explaining how the results can inform policy and practice effectively. Finally, the authors mention their plans to further validate the findings within a broader research project in Flanders, underscoring the commitment to thorough and credible research outcomes.

Thank you for addressing all the concerns. Congratulations!

*Dear reviewer, thank you for your feedback. We appreciate your positive evaluation and are glad that our adjustments are well received.*

Reviewer #3:

Please provide description of concept analysis and then the scoping review. Present their relationship with each other.

*Dear reviewer, thank you for your suggestion. Based on your feedback, we revised the Design section to first describe the concept analysis methodology, followed by the scoping review methodology, instead of the other way around. For each methodology, we added information about its purpose and limitations, to help clarify our rationale for combining them. This rationale for combining the methodologies is now also more explicitly stated in the final part of the section. (See also page 5, lines 92-108 of manuscript):*

*‘This review will use the methodological framework described by Lam Wai Shun and colleagues, which combines elements of both the concept analysis and the scoping review methodology [15]. These two methodologies will serve complementary purposes in our review.*

*The concept analysis methodology of Walker and Avant is a structured approach designed to explore and clarify concepts that are inconsistently used or lack conceptual clarity [14]. It offers structured guidelines for concept clarification through multiple steps: defining uses of the concept, defining attributes, antecedents, consequences, and empirical referents [14]. Nonetheless, this methodology does not specify a strategy for identifying relevant literature.*

*The scoping review methodology of Levac and colleagues [18] building upon the framework proposed by Arksey and O’Malley [19] provides a structured, transparent, and reproducible approach to systematically map the existing literature on a topic [18]. However, this methodology lacks specific guidelines for analyzing data for concept clarification.*

*By integrating these two methodologies, we can leverage the strengths of each individual methodology. The scoping review methodology ensures a systematic approach to identify and select relevant publications for our review, while the concept analysis methodology offers a structured framework for clarifying the concept of a ‘positive living environment’ in nursing homes [15]. Together they align with our goal of comprehensively exploring what constitutes a positive living environment in nursing homes.’*
